# Evidence of Genetic Instability in Tumors and Normal Nearby Tissues

**DOI:** 10.1371/journal.pone.0009343

**Published:** 2010-02-23

**Authors:** Giuseppe Geraci, Ida D'Elia, Rosanna del Gaudio, Rossella Di Giaimo

**Affiliations:** 1 Department of Biological Sciences, University of Naples Federico II, Napoli, Italy; 2 Ceinge Biotecnologie Avanzate s.c. a r.l., Napoli, Italy; Institute of Cancer Research, United Kingdom

## Abstract

**Background:**

Comprehensive analyses have recently been performed on many human cancer tissues, leading to the identification of a number of mutated genes but providing no information on the variety of mutations present in each of them. This information is of interest to understand the possible origin of gene mutations that cause tumors.

**Methodology/Principal Findings:**

We have analyzed the sequence heterogeneity of the transcripts of the human *HPRT* and *G6PD* single copy genes that are not considered tumor markers. Analyses have been performed on different colon cancers and on the nearby histologically normal tissues of two male patients. Several copies of each cDNA, which were produced by cloning the RT-PCR-amplified fragments of the specific mRNA, have been sequenced. Similar analyses have been performed on blood samples of two ostensibly healthy males as reference controls. The sequence heterogeneity of the *HPRT* and *G6PD* genes was also determined on DNA from tumor tissues. The employed analytical approach revealed the presence of low-frequency mutations not detectable by other procedures. The results show that genetic heterogeneity is detectable in *HPRT* and *G6PD* transcripts in both tumors and nearby healthy tissues of the two studied colon tumors. Similar frequencies of mutations are observed in patient genomic DNA, indicating that mutations have a somatic origin. *HPRT* transcripts show genetic heterogeneity also in healthy individuals, in agreement with previous results on human T-cells, while *G6PD* transcript heterogeneity is a characteristic of the patient tissues. Interestingly, data on *TP53* show little, if any, heterogeneity in the same tissues.

**Conclusions/Significance:**

These findings show that genetic heterogeneity is a peculiarity not only of cancer cells but also of the normal tissue where a tumor arises.

## Introduction

There is ample evidence that tumours arise as a consequence of genetic cell instability, which gives rise to an accumulation of mutated genes. The tumour phenotype is expected to appear when a critical number of transformation-related genes are damaged [1], [2], [3], [4]. Excluding tumours due to the effect of external factors such as viruses [5], stem cells appear to be good candidates for tumour origination because they are able to duplicate and have not reached a final and stable genetic stage and because their genomes must undergo reprogramming to express the proper differentiated phenotype. In line with this hypothesis is the finding that cultured tumour mouse erythroleukemia cells are particularly sensitive to mutations in specific periods during erythroid differentiation [6], [7], [8]. Random mutations are expected to occur more frequently in nucleotide positions that are not relevant for molecular functions and in genes not necessary to express a differentiated phenotype. Consequently, it is likely that a stem cell in which genetic instability arises does not lose the ability to undergo clonal expansion and to differentiate participating in the production of specific tissues. Under this assumption, genetic instability that is originated in a particular stem cell may produce, by clonal expansion, a number of cells in which further random mutations are likely to occur. It is also possible that the genetically unstable clones undergoing differentiation and final turnover may disappear from the tissue. During the period in which the genetically unstable stem cells are present and expand in the tissue, the probability increases that mutations may damage the critical transformation-related genes to produce a tumour phenotype. If this mechanism is operative in the case of spontaneous tumours, genes bearing mutations are likely to be observed not only in the tumours but also in nearby cells showing a histologically and functionally normal phenotype. Additionally, their genetic heterogeneity may be even higher than in tumour tissues. Data concerning mutations in HPRT and G6PD genes, analyzed here in two different colon tumours and nearby normal intestinal tissues of two male patients, lend support to this hypothesis.

## Materials and Methods

### Ethics Statement

All samples were collected with appropriate ethics approval: written informed consent for surgery to remove tumours and verbal consent to analyze them were provided by both patients and deposited at the Department of General Geriatric Oncological Surgery and Advanced Technology, School of Medicine, University of Naples Federico II. Reference blood samples were kindly provided by two male individuals of the Department of Biological Sciences, University of Naples Federico II, where this work was carried out.

### Patient Tissue Samples

Tumour tissues from two male Italian patients, a relapse of a colorectum carcinoma (patient A, born in 1937) and a primary colon sigma carcinoma (patient B, born in 1935), with corresponding samples of nearby intestinal tissues assessed to be histologically normal, were generously provided by the Department of General Geriatric Oncological Surgery and Advanced Technology, School of Medicine of the University of Naples Federico II. Different tissue fragments were removed from each sample to perform parallel independent analyses.

### Reference Control Samples for Genetic Heterogeneity

Blood samples of two ostensibly healthy male individuals, one 22 years old (C1) and a second 76 years old (C2), were used as reference controls of transcript heterogeneity to take into consideration possible differences due to age.

### Control Samples of Used Procedures

Two types of negative controls were used to check heterogeneity arising from experimental procedures: 1 - Bacterial clones containing a previously sequenced fragment of HPRT and G6PD cDNA that were amplified, cloned and sequenced; 2- HPRT and G6PD cDNA obtained from a human male CaCO-2 cell culture that were amplified, cloned and sequenced.

### Preparation of Clones of Gene Transcripts

Total RNAs from tumour and healthy tissues of two patients and from the blood of two reference individuals were prepared using an SV Total RNA isolation kit (Promega, USA). cDNA preparations were obtained using Superscript III and random primers (Invitrogen, California). Both procedures were carried out according to the respective manufacturers' protocols. RT-PCR amplifications of individual cDNAs were performed using specific oligonucleotides for HPRT (NM_000194) and G6PD (NM_000402.3). Also, TP53 (NM_000546) cDNA was amplified to monitor genetic heterogeneity in a gene known to be involved in tumours. Specifically, HPRT cDNA was amplified from nucleotide position 16 to 526 using oligonucleotides forward CCTGGCGTCGTGATTAG and reverse GCTTATATCCAACACTTCGTGG; G6PD cDNA was amplified from nucleotide position 159 to 561 using oligonucleotides forward CGATGCCTTCCATCAGTCG and reverse GGACTCGTGAATGTTCTTGG; TP53 cDNA was amplified from nucleotide position 427 to 942 using oligonucleotides forward GTGCAGCTGTGGGTTGATTCC and reverse GGAGCTGGTGTTGTTGGGCAG. Numerations start from nucleotide A of ATG initial triplets. PCR cycle conditions were initially 5 min at 94°C, followed by cycles of 1 min at 94°C, 50 sec at 55°C, 50 sec at 72°C, and was repeated 35 times followed by 10 min at 72°C. The annealing temperature was 58°C for HPRT amplifications. Clones were amplified in 3 different PCR experiments from 3 different cDNA preparations from each sample of tumour and healthy tissues of patients and from reference and control samples using Taq DNA polymerase (SIGMA, USA) or Expand High Fidelity (Roche, USA), with similar results. Amplified cDNA fragments were isolated by agarose gel electrophoresis, purified by NucleoSpin Extract II (Macherey-Nagel, Germany) and cloned using a PCR cloning Kit (Qiagen, Germany). Individual clones were randomly chosen and sequenced (MWG, Germany). The DNA sequences of most clones showing mutations were confirmed by a different sequencing unit (Zoological Station of Naples, Italy). Nucleotide sequences were analyzed using Blast and ClustalW algorithms.

### Preparation of Clones of Genomic Regions

Genomic DNA was isolated with the Trizol reagent (Invitrogen, California) from tumour tissues of patient A, which were also used for RNA extraction. Exons 3, 4 and most of exon 5 (204/218 bp) of the G6PD gene (ENST00000291567) were amplified by PCR as a single amplicon of 1124 bp (total length of exons, 351 bp) by designing the forward oligonucleotide in intron 1 (CAAGACAGACATGCTTGTGG) and the reverse oligonucleotide in exon 5 (GGACTCGTGAATGTTCTTGG). Exons 2, 3, 4 and 6 of the HPRT gene (ENST00000298556) were amplified separately by designing each oligonucleotide in the respective flanking intron regions, taking care to produce segments of similar lengths. HPRT exon amplifications were performed using the following pairs of oligonucleotides, forward and reverse, respectively: CGAACTCCTGAGCTCAGGCAG and GTTCTGGTCCCTACAGAGTCC for exon 2 (amplicon length 573 bp, exon length 107 bp); TGTATTGCCCAGGTTGGTG and CAAGTCCCAACAGCAATTCC for exon 3 (amplicon length 540 bp, exon length 184 bp); CAGTAATGGCCGATTAGGAC and ACCTAGACTGCTTCCAAGGG for exon 4 (amplicon length 457 bp, exon length 66 bp); GCTGTCATTGATCCTGCACC and CTCTGCCATGCTATTCAGGAC for exon 6 (amplicon length 569 bp, exon length 83 bp).

PCR cycle conditions for HPRT exons 3, 4 and 6 were initially 5 min at 94°C, followed by 40 cycles of 50 sec at 94°C, 35 sec at 58°C, 45 sec at 72°C and finally 10 min at 72°C with the following exceptions: the elongation time of G6PD was 70 sec at 72°C and the annealing temperature was 62°C for HPRT exon 2. Amplified DNA fragments obtained from 3 different parallel PCR experiments were pooled and purified by NucleoSpin Extract II (Macherey-Nagel, Germany) and cloned using a PCR cloning Kit (Qiagen, Germany). Individual clones were randomly chosen and their insert DNAs were sequenced (MWG, Germany). Nucleotide sequences were analyzed using Blast and ClustalW algorithms.

### Statistical Analyses

Student t-distribution analyses were performed using an Excel worksheet.

### Frequency of Mutation

The frequency of mutations reported in this manuscript was calculated for each sample with this formula: Total number of detected mutations/(base pair length of the amplified fragments × number of sequenced clones) × 1000, considering that all analyzed samples were produced with the same number of PCR amplification cycles.

## Results

### Transcript Analysis on Patient Tissue Samples

The single point mutations identified on the HPRT, G6PD and TP53 transcripts are reported in Table 1, Table 2 and Table 3, respectively. Analyses were performed by cloning and sequencing the PCR-amplified segments of the cDNAs of the colon tumours and nearby healthy tissues of patient A, bearing a tumour relapse, and patient B, bearing a primary tumour. It should be noted that the HPRT and G6PD genes are present in a single copy on the single X chromosome of human male cells and their nucleotide sequence should be only one. In humans, G6PD is present only on the X chromosome, while additional HPRT gene fragments are present in several positions on other chromosomes, but none of those fragments is expressed and, moreover, each would be easily identified because of a distinctly different composition in the sequenced region [9].

**Table 1:** Mutations in HPRT transcripts in tumour and nearby healthy tissues.

Patient and Tissue Type	Mutations/clone	Nucleotide position (mutation)	Number of identical clones
A Healthy	1	**66(T→C)°**	1
A Healthy	1	**150(T→C)**	3
A Healthy	1	221(T→C)	1
A Healthy	1	**386(A→G)**	5
A Healthy	1	**391(T→A)#**	1
A Healthy	1	446(T→C)	1
A Healthy	1	**447(G→T)°**	1
A Healthy	1	490(C→T)	1
A Healthy	2	185(T→C) 274(T→C)	1
A Healthy	3	87(T→C) 190(G→A) 386(A→G)	1
A Healthy	0	*wt*	24/40 total clones
A Tumour	1	36(T→C)	1
A Tumour	1	**66(T→C)°**	1
A Tumour	1	100(A→G)*	2
A Tumour	1	119(G→A)	1
A Tumour	1	146(T→C)	1
A Tumour	1	245(T→C)	1
A Tumour	1	363(T→C)	1
A Tumour	1	414(C→T)	1
A Tumour	1	**447(G→T)°**	1
A Tumour	1	473(T→C)	1
A Tumour	2	193(C→T) 485(G→A)	1
A Tumour	2	81(T→C) 427(A→G)	1
A Tumour	2	112(C→T) 343(A→G)	1
A Tumour	2	270(T→C) 330(A→G)	1
A Tumour	2	**231(C→T) 299(T→C)**	2
A Tumour	5	**200(T→A) 223(T→C)# 278(T→C) 447(G→A) 486(C→T)**	2
A Tumour	6	153(A→C) 198(T→C) 355(G→A) 419(G→A) 431(A→G) 496(A→G)	1
A Tumour	0	*wt*	16/36 total clones
B Healthy	1	33(T→C)	1
B Healthy	1	59(A→G)	1
B Healthy	1	331(A→G)	1
B Healthy	1	400(G→A)	1
B Healthy	2	124(A→G) 388(G→A)	1
B Healthy	2	**223(T→C)#** 227(C→T)	1
B Healthy	2	**308(A→G) 323(A→G)**	2
B Healthy	0	*wt*	14/22 total clones
B Tumour	1	206(A→G)	1
B Tumour	1	**215(A→G)**	3
B Tumour	1	**391(T→A)#**	1
B Tumour	2	120(A→G) 147(T→C)	1
B Tumour	4	57(T→C) 100(A→G)* 302(G→A) 315(T→A)	1
B Tumour	0	*wt*	16/23 total clones

Mutations present in multiple clones in the same tissues are reported in bold-type, those present in different tissues are underlined. °, mutation in healthy and tumour tissues of the same patient; *, mutation in the tumour tissues of patients A and B; #, mutation in both the healthy tissues of patient A and the tumour tissues of patient B. Numeration of nucleotide positions starts from A of the initial ATG triplet. *wt*: wild type sequence.

**Table 2:** Mutations in G6PD transcripts in tumour and nearby healthy tissues.

Patient and Tissue Type	Mutations/clone	Nucleotide position (mutation)	Number of identical clones
A Healthy	1	276 (C→T)	1
A Healthy	1	301 (G→A)	1
A Healthy	1	**484 (T→C)°**	1
A Healthy	1	494 (A→G)	1
A Healthy	1	**503 (T→C)+**	1
A Healthy	1	510 (G→A)	1
A Healthy	1	526 (G→A)	1
A Healthy	2	199 (A→G) 468 (T→C)	1
A Healthy	2	**261 (G→A) 300 (T→C)**	2
A Healthy	0	*wt*	17/27 total clones
A Tumour	1	182 (C→T)	1
A Tumour	1	188 (T→C)	1
A Tumour	1	218 (T→C)	1
A Tumour	1	289 (A→C)	1
A Tumour	1	361 (A→G)	1
A Tumour	1	392 (T→C)	1
A Tumour	1	413 (T→C)	1
A Tumour	1	**484 (T→C)°**	1
A Tumour	0	*wt*	17/25 total clones
B Healthy	1	185 (A→C)	1
B Healthy	1	333 (C→T)	1
B Healthy	1	401 (G→A)	1
B Healthy	1	425 (A→G)	1
B Healthy	1	459 (C→T)	1
B Healthy	1	**503 (T→A)+**	1
B Healthy	1	**541 (A→T)°**	1
B Healthy	2	229 (A→T) 370 (G→A)	1
B Healthy	2	259 (C**→**G) 487 (C→T)	1
B Healthy	2	278 (A→G) 522 (G→A)	1
B Healthy	0	*wt*	21/31 total clones
B Tumour	1	251 (G→A)	1
B Tumour	1	310(C→T)	1
B Tumour	1	430 (G→A)	1
B Tumour	1	465 (G→A)	1
B Tumour	1	467 (A→G)	1
B Tumour	1	**541 (A→G)°**	2
B Tumour	0	*wt*	17/24 total clones

Mutations present in multiple clones in the same tissues are reported in bold-type, those present in different tissues are also underlined. °, mutation in healthy and tumour tissues of the same patient, +, mutation in the healthy tissues of both patients A and B. Numeration of nucleotide positions starts from A of the initial ATG triplet. *wt*: wild type sequence.

**Table 3:** Mutations in TP53 transcripts in tumour and nearby healthy tissues.

Patient and Tissue Type	Mutations/clone	Nucleotide position (mutation)	Number of identical clones
A Healthy	1	536 (A→T)	1
A Healthy	1	628 (A→G)	1
A Healthy	2	535 (C→T) 874 (A→G)	1
A Healthy	2	647 (T→C) 681 (T→C)	1
A Healthy	2	655 (C→T) 915 (G→A)	1
A Healthy	0	*wt*	10/15 total clones
A Tumour	1	461 (G→A)	1
A Tumour	1	545 (G→A)	1
A Tumour	2	574 (C→T) 789 (T→C)	1
A Tumour	2	543 (C→T) 635 (T→C)	1
A Tumour	0	*wt*	7/12 total clones
B Healthy	0	*wt*	12/12 total clones
B Tumour	1	631 (A→G)	1
B Tumour	1	831 (T→C)	1
B Tumour	0	*wt*	8/10 total clones

Numeration of nucleotide positions starts from A of the initial ATG triplet. *wt*: wild type sequence.

HPRT transcripts, proposed as a probe of gene instability in human cells [10], [11], [12], have shown the largest variety of mutations also in the present work. A total of 121 HPRT clones were sequenced and 51 of them showed mutations (Table 1). In particular, the highest percent value of mutated HPRT clones (55%) was found in the carcinoma relapse of patient A (20 mutated in 36 sequenced clones). A similar genetic heterogeneity resulted from the healthy tissues of patient A and from the healthy and tumour tissues of patient B (40%, 36% and 30% mutated clones, respectively). The high number of point mutations observed in some HPRT clones, up to 6 (Table 1), is difficult to rationalize on the basis of frequency of errors derived from cell division. As already suggested [10], this high number might indicate the activity of a mutator phenotype. Significantly, clones with identical single point mutations were present in healthy and tumour tissues of patients A and B.

Data concerning heterogeneity in G6PD transcripts showed similar percent values of mutated clones in the cancer and healthy tissues of both patients A and B, with about 34% average value (Table 2). In the 107 sequenced clones, 35 showed mutations. Single point mutations, as observed for HPRT gene transcripts, were present in both tumour and healthy tissues. Furthermore some clones were found bearing mutations at the same sequence position, in some cases a transition and in others a transversion. For example, 1 clone of the healthy tissue of patient A showed the transition T>C at nucleotide 503, whereas 1 clone of the healthy tissue of patient B shows the transversion T>A.

Data concerning TP53 transcripts, analyzed here as a gene generally correlated to tumours, involved a lower number of clones. In humans, transcripts of this gene present on chromosome 17 might show difference(s) due to heterozygosity, but all of the analyzed clones of the healthy tissues of patient B showed the identical normal sequence. The corresponding tumour tissues showed a value of percent mutated clones (Table 3) that was too low to be indicative of heterogeneity in the procedure used here, particularly considering the results reported below on control samples and the small number of analyzed clones. Interestingly, in both healthy and tumour tissues of patient A, bearing a colorectum carcinoma relapse, all observed single point transitions in TP53 transcripts, with the exception of mutations at position 628 (A>G) and 789 (T>C), have been reported in a different tumour [IARC TP53 mutation database, 13].

### Reference and Control Samples

#### HPRT transcripts

**Figure 1:**
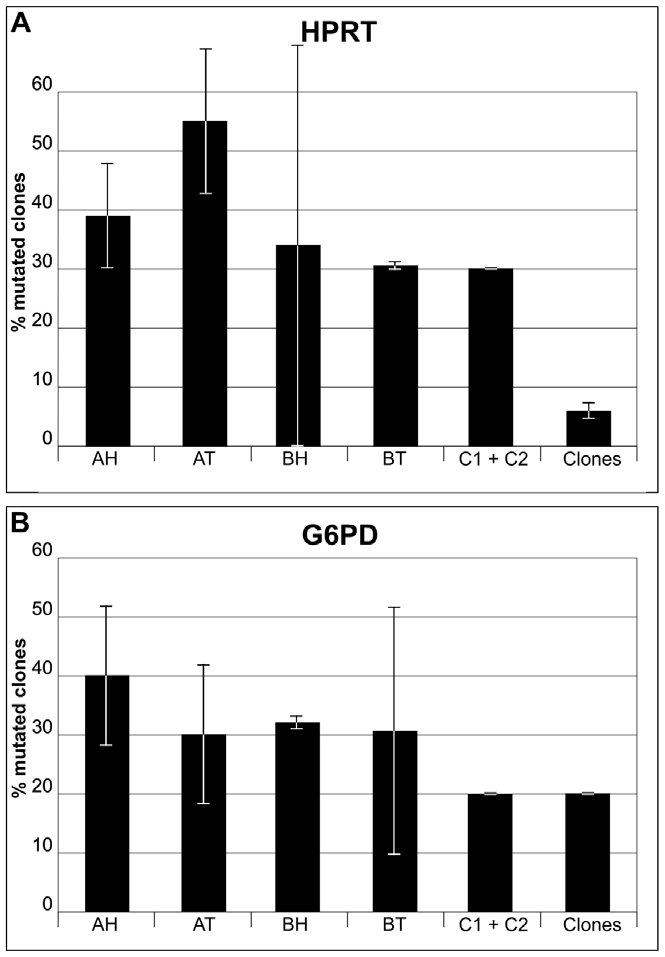
Standard deviations of percent mutated clones of HPRT and G6PD cDNAs. Analyses of 3 independent experiments for each gene on tumour and healthy tissues of patients A and B, on reference samples C1 and C2, and on control clones. Panel A, values of HPRT transcripts; Panel B, values of G6PD transcripts. AT and BT, values of clones of Tumour tissues; AH and BH, values of clones of Healthy tissues nearby tumours. Clones, values of control clones.

**Figure 2:**
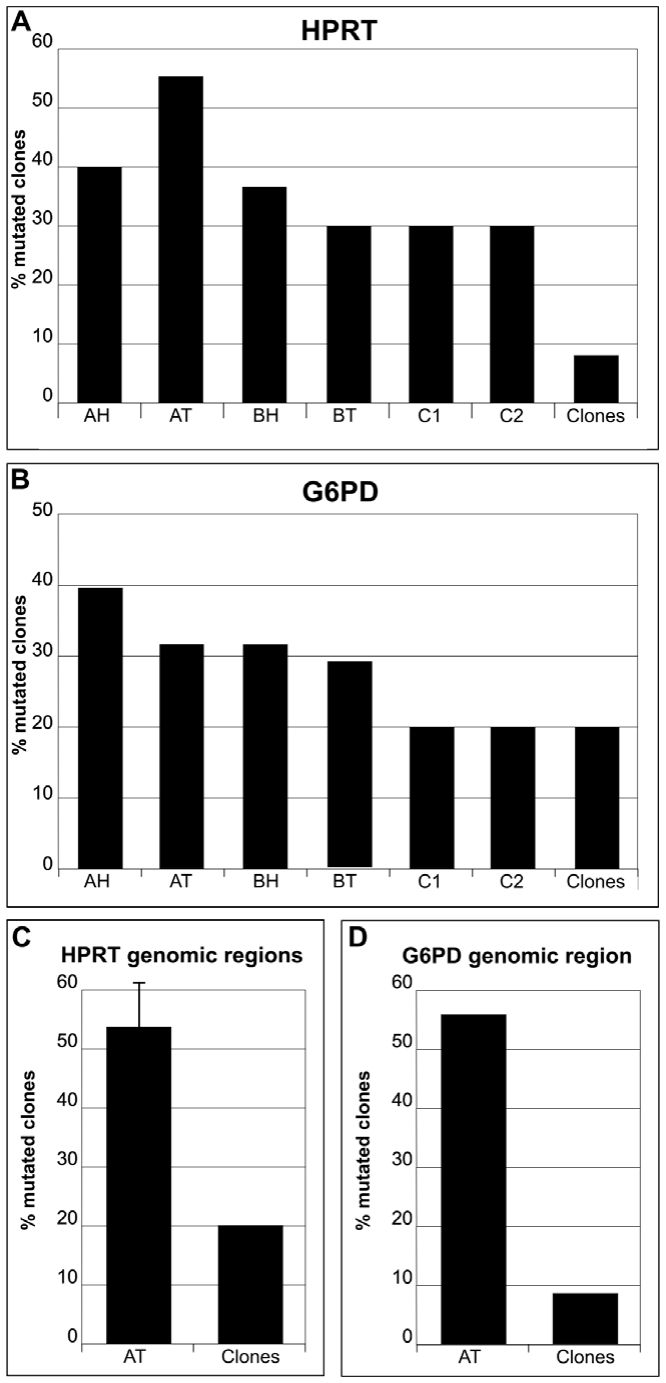
Percent mutated clones of HPRT and G6PD cDNA and genomic regions. Panel A, values of HPRT transcripts; Panel B, values of G6PD transcripts. Panel C, values of HPRT genomic regions; Panel D, values of G6PD genomic regions. Percent values have been calculated on pooled data from each independent analysis. AT and BT, values of clones of Tumour tissues of patients A and B, respectively; AH and BH, values of clones of Healthy tissues near tumours. C1 and C2, values of reference samples. Clones, values of control samples.

Reference control analyses for heterogeneity of HPRT transcripts were carried out with the same experimental approach by sequencing 10 clones from each blood sample of C1 and C2 ostensibly healthy individuals. The results on C1, a 22 year old male, showed 1 clone with a single point mutation and 2 clones with 2 point mutations, all different from each other. Similar analyses on C2, a 76 year old male, showed 3 clones with single point mutations, all different from each other and from those observed in the tumour and nearby healthy tissues of the two patients analyzed here. Errors due to the analytical procedure were evaluated by sequencing 42 control clones obtained from an already cloned and sequenced HPRT segment. A 7% value of mutated clones resulted as error due to the procedure (Fig. 1A and 2A).

#### G6PD transcripts

Reference control analyses for G6PD transcript heterogeneity on healthy individuals, as for HPRT analyses, were performed on 10 clones of both C1 and C2 blood cDNA samples. 10 cDNA clones were also prepared from the human colon carcinoma cell line CaCO-2 and analyzed as controls. In both cases, 2 clones out of 10 showed mutations. G6PD transcripts from CaCO-2 cells showed a clone with a single point mutation and another with 2 point mutations. In total, 6 clones out of 30 were mutated, corresponding to a 20% value. The analytical procedure, tested on 20 clones obtained from PCR amplification and cloning of an already sequenced G6PD fragment, showed 4 mutated clones, 2 of which were identical, corresponding to 20% mutated clones (Fig. 1B and 2B).

### Statistical Analysis on Transcript Samples

Percent mutated clones relative to the 3 series of independent experiments performed for HPRT (Panel A) and G6PD (Panel B) with respective standard deviations are reported in Figure 1. It is apparent from the comparison between the standard deviation values that the cancer and nearby healthy tissues were heterogeneous materials, while the reference samples and control clones were homogeneous. Statistical analyses with the student's t-test were performed comparing, for each gene, percent values of total mutated clones from tumour and healthy tissues of patient A and B samples versus percent of total mutated control clones (Fig. 2). The results show that the values of genetic heterogeneity found in cancer and histologically healthy tissues of the two patients for both HPRT (Fig. 2A) and G6PD (Fig. 2B) transcripts are statistically similar to each other and different from the controls. Specifically, for HPRT transcripts, the percent mutated clones in the 121 sequenced for patient A and B healthy and cancer tissues, analyzed versus percent values of 62 control clones, resulted in p = 0,0066, while the student's t-test on patient A versus patient B resulted in p = 0,2. Similar analyses for 107 total G6PD clones versus 50 control samples resulted in p = 0,0021. No statistical difference resulted comparing patient A and patient B values (p = 0,25).

### Genomic Analysis on Patient Tissue Samples

To get some insights into the mechanism(s) underlying the genetic heterogeneity found in HPRT and G6PD mRNA sequences, we analyzed the genomic DNA fragments corresponding to the transcript regions under investigation with the same experimental approach. We performed these analyses on genomic DNA from the tumour tissues of patient A that showed the highest percent of mutated clones. The single point mutations identified on the HPRT and G6PD genomic regions are reported in Table 4 and Table 5, respectively. HPRT exons 2, 3, 4 and 6 were amplified in 4 different genomic segments due to the lengths of the introns, while G6PD exons 3, 4 and 5 were amplified in a single genomic fragment. In particular, for HPRT sequence analyses (Table 4), 25 clones out of 60 showed mutations occurring in exon and intron regions. In detail, 6 clones showed single point mutations in exons 3 and 4, and four of these clones had the same A>G transition at nucleotide 239; one clone showed 2 point mutations in exon 2, and 1 of these, at nucleotide 57, was already identified in HPRT transcript analysis on tumour tissues of patient B. No clone showed mutations in exon 6. Other mutations were in intron regions.

**Table 4:** Mutations in HPRT gene regions in tumour tissues of patient A, colorectum carcinoma relapse.

Number of clones	Exon 2 (107 bp)	Exon 3 (184 bp)	Exon 4 (66 bp)	Exon 6 (83 bp)	Number of Mutations in Introns
1	45 (A→G) 57 (T→C)*				4
1					3
1					2
3					1
9/15 total clones	*wt*				*wt*
4		239 (A→G)			
2					2
2					1
7/15 total clones		*wt*			*wt*
1			374 (T→C)		1
1			320 (A→G)		
3					1
10/15 total clones			*wt*		*wt*
6					1
9/15 total clones				*wt*	*wt*

Numeration of nucleotide positions of exons starts from A of the initial ATG triplet of the coding sequence. *, mutation already identified in cDNA samples from tumour tissues of patient B. *wt*: wild type sequence.

**Table 5:** Mutations in the G6PD gene region in tumour tissues of patient A, colorectum carcinoma relapse.

Number of clones	Exon 3 (38 bp)	Exon 4 (109 bp)	Exon 5 (204 bp)	Number of Mutations in Introns
1		254 (T→C) 335 (A→G)		
2		329 (T→C)		2
1			418 (G→A)	
1			464 (T→C)	
1			531 (C→T)	
4				1
1				2
9/20 total clones	*wt*	*wt*	*wt*	*wt*

Each clone was sequenced on both filaments. Numeration of nucleotide positions of exons starts from A of the initial ATG triplet of the coding sequence. *wt*: wild type sequence.

Data concerning G6PD genomic regions (Table 5) showed that 11 clones out of the 20 sequenced displayed mutations; 5 clones showed single point mutations in exons 4 and 5, and two of these clones had an identical T>C transition at nucleotide 329; one clone showed 2 point mutations in exon 4. No clone showed mutations in exon 3. Other mutations were in intron regions.

The percent values of mutated clones of HPRT (Fig. 2C) and G6PD (Fig. 2D) genomic regions are reported in Figure 2. These two genes show similar genetic heterogeneity because, in both cases, 50% mutated clones were observed. The average value of percent mutated clones of each analyzed HPRT genomic fragment is reported with the standard deviation (Fig. 2C).

**Figure 3:**
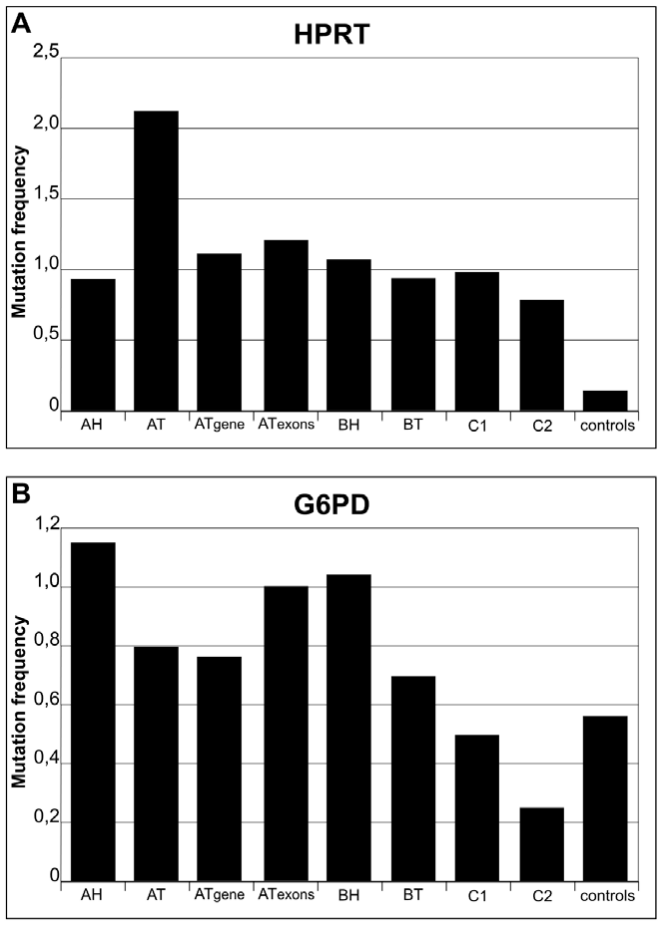
Mutation frequency values of all analyzed sequences of HPRT and G6PD cDNA and genes. Panel A, values of HPRT sequences; Panel B values of G6PD sequences. AH, AT, BH, BT: values calculated for cDNA clones of Tumour and nearby Healthy tissues of patients A and B. C1 and C2: values for cDNA clones from blood tissues of 2 healthy reference individuals. AT gene: values for clones of global genomic regions of DNA from tumour tissues of patient A. AT exons: values for the exon regions of the sequenced genomic regions. Controls: values for clone samples.

To compare the results on G6PD and HPRT genes to their transcripts in all of the analyzed samples, the frequencies of mutation were calculated considering the number of sequenced clones and the lengths of each amplified fragment (Fig. 3). The genomic mutation frequencies are reported both for exons and for the entire sequenced DNA regions, including introns. The results on the HPRT gene (Fig. 3A) show that the values of mutation frequency of reference C1 and C2 transcripts are statistically different from the control samples (p = 0.006). No significant difference was observed when comparing the cDNA mutation frequencies of C1 and C2 reference controls, patient A healthy tissues and patient B tumour and nearby healthy tissues with mutation frequencies of the respective genomic sequences (p>0.05). Overall, these data indicate that heterogeneity found in HPRT cDNA clones has a somatic origin. HPRT heterogeneity appears to be independent of age (22 and 76 years), health, cancer stage and tissue type (intestinal tissues and blood). Interestingly, the mutation frequency of transcripts in the tumour tissues of patient A differed from the others and, in particular, from the mutation frequencies of its corresponding genomic regions. This suggests that, in this case, mutations are introduced during processes leading to mRNAs.

The results on the G6PD gene (Fig. 3B) show that the mutation frequency values of the C1 and C2 reference samples were not different from controls (p>0.05), indicating that healthy people, regardless of age, show no significant genetic heterogeneity in this gene. However, the values of the G6PD mutation frequency of reference healthy individuals were statistically different from those of the transcripts and of the corresponding genomic regions of both patients (p = 0.009), indicating that G6PD genetic heterogeneity is specific of cancer and nearby healthy tissues in the two studied colon tumours.

## Discussion

The results presented here provide evidence of a similar value of genetic heterogeneity in HPRT and G6PD transcripts in cells of colon carcinoma and nearby healthy tissues in the two studied patients. The mutation frequencies of the HPRT and G6PD gene sequences of patient A were found to be not statistically different from the corresponding values of the cDNAs. This indicates that the mutations are present in genes and not introduced in the steps that produce mRNAs. Only mutation frequencies of cDNA of the colorectum carcinoma relapse were higher than the corresponding value at the DNA level, suggesting the occurrence, in this case, of additional mechanism(s) that produce mutations in the processes leading to mRNAs.

It is interesting to note that the standard deviation values of percent mutated clones of patient tissues were dramatically different from the corresponding values of the two reference blood samples of healthy individuals and from the control clones (Fig. 1). This suggests that patient tissues are heterogeneous materials, in line with the hypothesis of cellular genetic heterogeneity in tumours [14], while healthy reference samples and controls appeared to be homogeneous, as expected.

In agreement with previous data on the DNA of circulating T-lymphocytes [10], [11], [15], [16], our data clearly show genetic heterogeneity for HPRT in healthy individuals (Fig. 3A). The present work, however, provides evidence that HPRT heterogeneity is present not only in blood samples but also in intestinal colon tissues with similar values, indicating that this genetic heterogeneity is a characteristic of the gene and is not a consequence of genomic rearrangements occurring in a particular tissue. Conversely, the heterogeneity found for the G6PD gene and transcripts in reference healthy individuals had values significantly lower than those of patient tissues and similar to those of control clones (Fig. 3B), showing that heterogeneity in this gene is a peculiarity of the tumour and the nearby healthy tissues.

The observed high percent of mutated cDNA clones (Fig. 2A and B) should occur during the life span of the cell because only about 1×10^−8^ base pairs are incorporated as erroneous nucleotides per cell division [17]. DNA mutations are reported to be produced in other functional steps, such as the transcription-coupled repair process [18] that has been proposed to be responsible for SNP in human genes [19]. The different frequencies of gene mutations observed here could depend on different gene expression levels and/or on local chromatin organization [20], [21], [22], [23]. However, other mechanisms have been proposed through which epigenetic factors might give rise to localized nucleotide changes in DNA composition. Environmentally induced changes in the activity of chromatin-modifying enzymes can lead to changes in DNA sequences. If these changes occur in somatic cells, the resulting mutations might be important in triggering disease states [reviewed in 24].

Recently, it has been documented that a tumour may derive from one single genetically unstable cell [25]. Our data show that several unstable cells seem to be present in normal tissues where a tumour arises. It should be noted that the genetic heterogeneity here found might not be revealed if sequence analyses are carried out directly on the products of PCR amplification. This is apparent considering that in the experiments presented here, a minimum of 45% of clones were found with the same wild type sequence, while a maximum of 5 clones out of 40 were found with an identical mutated position (HPRT transcripts showing (A>G) at nucleotide position 386 in tumour tissue of patient A), corresponding to 15% relative abundance (Table 1). This percent value has no appreciable effect on the amplitudes of the sequential pattern of bands produced by the analyzing platforms that detect the more abundant nucleotides.

It is known [26], and we found here, that the procedure based on sequencing several individual clones is affected by possible errors due to the amplification step by Taq DNA polymerase. This enzyme introduces erroneous bases with a statistical frequency of about 1 every 5000 polymerized nucleotides. To compensate for this error, we performed all PCR amplification reactions rigorously under the same experimental conditions using an identical number of cycles. In this way, the frequency of mutation of samples and controls can be directly compared to evaluate background error contribution. The results reported here on control samples show a frequency of error spanning from 1/2000 (G6PD control clones) to 1/5000 (HPRT control clones), depending on the sequence composition, possibly because of the known nearest neighbour nucleotide effect on the accuracy of replication [27], [28]. Results on the isolation of mutated clones by amplification of the particular variants directly on the cDNA samples using modified oligonucleotides [29, and references therein] are not presented here because low levels of false positives were generated. For this reason, we decided to avoid any screening criterion of the data and analyze them comparing the frequency of erroneous inserted nucleotides as determined on the basis of the number and lengths of the sequenced clones. This analytical approach revealed values of TP53 mutation frequency, evaluated on the 49 analyzed clones, that were slightly above the largest error value determined in control experiments. This indicates that the single nucleotide mutations, observed here and reported in the TP53 mutation database [13], might really correspond to the background genetic noise, and not be specifically correlated to tumour formation. The low mutation frequency observed for TP53 on sample tissues where other genes show higher values confirms that genes may be differently affected by background genetic noise. The possibility that the source of most of the mutations presented here might depend on the reverse transcriptase infidelity is not apparent in our results. Indeed, G6PD heterogeneity was similar for C1 and C2 reference samples and for control clones (Fig. 2B and 3B). However, C1 and C2 RNA samples were reverse-transcribed and then amplified by PCR, while control clone DNA samples were only PCR-amplified. The reverse-transcription step did not add any appreciable increase in either percent mutated clones (Fig. 2B) or frequency of mutation values (Fig. 3B).

Overall, the results reported here appear relevant to a mechanism of spontaneous tumour formation by providing information on the variety of mutations occurring in a gene, and by complementing the results of massive global genomic analyses performed on tumour tissues [30], [31]. Our finding of a similar genetic heterogeneity in colon cancer tissues, nearby healthy tissues and in reference healthy samples for the HPRT gene is also an indication that some genes have physiologically low levels of mutations. Normally, this heterogeneity may have no functional effects and escape detection with commonly used analytical procedures. It was revealed here because the cumulative abundance of each single nucleotide substitutions results in a number of mutated clones, each randomly representing the variety of mutations occurring in the analyzed tissues, which can be detected by the cloning procedures. When the spontaneous low background genetic heterogeneity increases and hits relevant genes, spontaneous tumours may arise. This implies that no general patterns of gene mutation and no sequential set of gene modifications are expected, although each tumour, depending on its type and tissue of origin, may present more frequent mutations in the particular genes that are active in the particular differentiated tissue.

Our approach, based on comparing genetic heterogeneity in tumour and nearby healthy tissues, if confirmed by further analyses on other genes, may help to distinguish spontaneous and induced tumours and may be useful for tumour follow-up.
